# Supplementary material for: Association between oral microbiome and seven types of cancers in East Asian population: a two-sample Mendelian randomization analysis
Source: Front Mol Biosci. 2023 Nov 21;10:1327893. doi: 10.3389/fmolb.2023.1327893 (PMC10702768; doi:10.3389/fmolb.2023.1327893)
Supplement: Supplementary file 1 [file DataSheet1.ZIP › Supplementary Materials/MR plots of saliva/saliva═╝/Pancreatic cancer/pheno.1087_to_pancreatic cancer_forest.pdf]

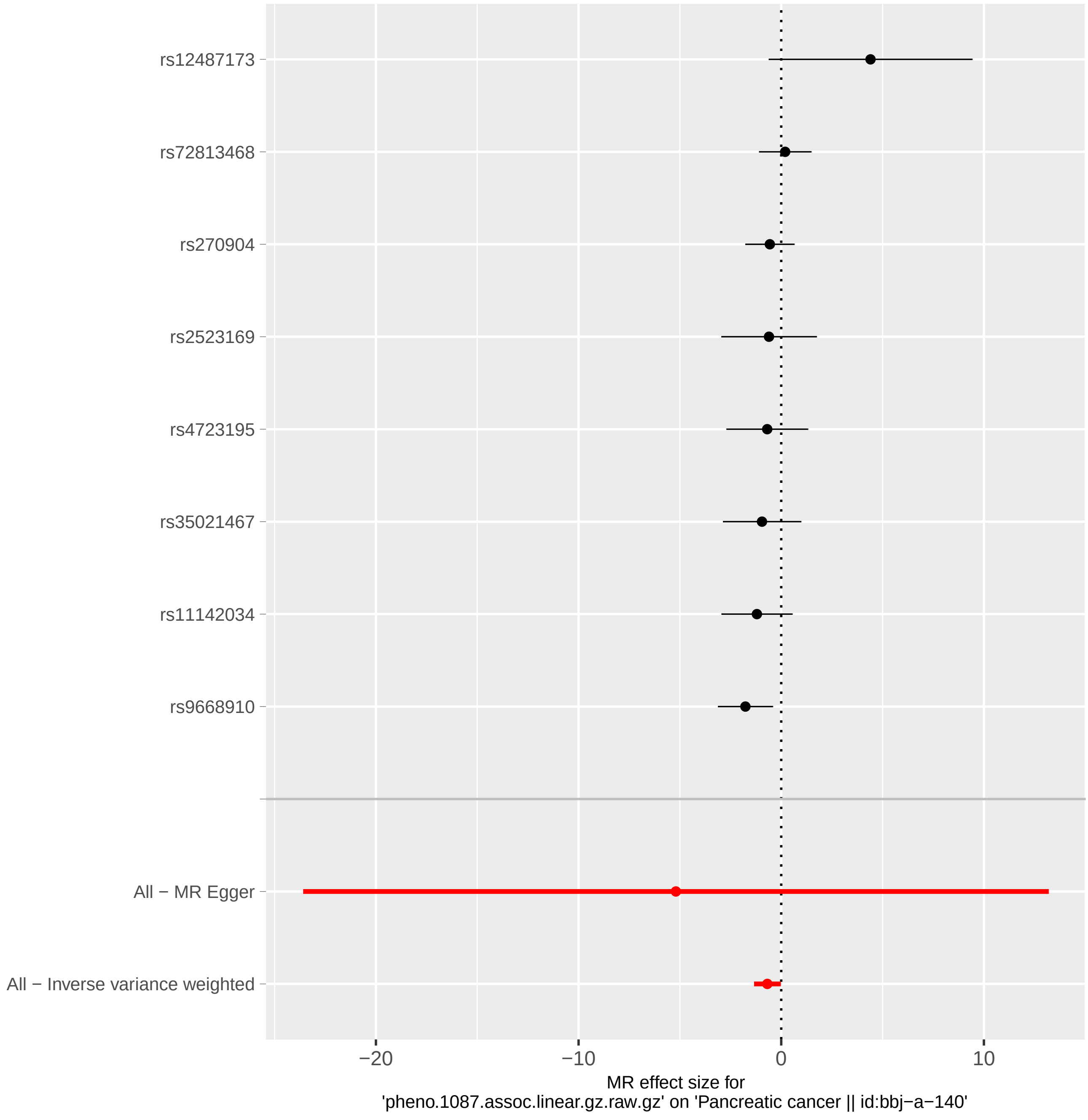
rs12487173
rs72813468
rs270904
rs2523169
rs4723195
rs35021467
rs11142034
rs9668910
All − MR Egger
All − Inverse variance weighted
−20
−10
0
10
MR effect size for
'pheno.1087.assoc.linear.gz.raw.gz' on 'Pancreatic cancer || id:bbj−a−140'
